# Hydrogen Production from Chemical Hydrides via Porous Carbon Particle Composite Catalyst Embedding of Metal Nanoparticles

**DOI:** 10.3390/mi16020172

**Published:** 2025-01-31

**Authors:** Sahin Demirci, Osman Polat, Nurettin Sahiner

**Affiliations:** 1 Department of Food Engineering, Faculty of Engineering, Istanbul Aydin University, Florya Halit Aydin Campus, Istanbul 34295, Turkey; sahindemirci@gmail.com; 2 Department of Chemical and Biomolecular Engineering, University of South Florida, Tampa, FL 33620, USA; o.polat1996@googlemail.com; 3 Department of Chemistry, Faculty of Sciences, Canakkale Onsekiz Mart University, Terzioglu Campus, Canakkale 17100, Turkey; 4 Department of Ophthalmology, Morsani College of Medicine, University of South Florida, 12901 Bruce B. Downs Blvd, MDC21, Tampa, FL 33612, USA; 5 Department of Bioengineering, U.A. Whittaker College of Engineering, Florida Gulf Coast University, Fort Myers, FL 33965, USA

**Keywords:** carbon-based catalyst, carbon–metal composite catalyst, hydrolysis of sodium borohydride, ammonia borane, hydrogen production, renewable energy

## Abstract

Porous carbon particles (PCPs) prepared from sucrose via the hydrothermal method and its modified forms with polyethyleneimine (PEI) as PCP-PEI were used as templates as in situ metal nanoparticles as M@PCP and M@PCP-PEI (M:Co, Ni, or Cu), respectively. The prepared M@PCP and M@PCP-PEI composites were used as catalysts in the hydrolysis of NaBH_4_ and NH_3_BH_3_ to produce hydrogen (H_2_). The amount of Co nanoparticles within the Co@PCP-PEI structure was steadily increased via multiple loading/reducing cycles, e.g., from 29.8 ± 1.1 mg/g at the first loading/reducing cycles to 44.3 ± 4.9 mg/g after the third loading/reducing cycles. The Co@PCP-PEI catalyzed the hydrolysis of NaBH_4_ within 120 min with 251 ± 1 mL H_2_ production and a 100% conversion ratio with a 3.8 ± 0.3 mol H_2_/(mmol cat·min) turn-over frequency (TOF) and a lower activation energy (Ea), 29.3 kJ/mol. In addition, the Co@PCP-PEI-catalyzed hydrolysis of NH_3_BH_3_ was completed in 28 min with 181 ± 1 mL H_2_ production at 100% conversion with a 4.8 ± 0.3 mol H_2_/(mmol cat·min) TOF value and an Ea value of 32.5 kJ/mol. Moreover, Co@PCP-PEI composite catalysts were afforded 100% activity up to 7 and 5 consecutive uses in NaBH_4_ and NH_3_B_3_ hydrolysis reactions, respectively, with all displaying 100% conversions for both hydrolysis reactions in the 10 successive uses of the catalyst.

## 1. Introduction

Improvements in human welfare and health resulting from industrial progress has led to increased energy consumption, resulting in lateral effects on climate change and therefore increased demand for more sustainable and greener energy sources to counteract fossil fuel-associated problems [1,2,3,4]. Currently, the world’s primary source of hydrogen (H_2_) is mostly from fossil fuels. For example, at the end of 2021, it was found that 47% of hydrogen comes from natural gas, 27% from coal, 22% from oil (as a by-product), and only 4% from water via electrolysis [5,6]; however, there is considerable potential for H_2_ to evolve into a sustainable energy resource through the adoption of new and alternative sources. Current advancements have identified non-fossil fuel-based hydrides as viable candidates for H_2_ production, addressing the challenges associated with traditional fossil fuel reliance [7,8,9]. Consequently, the development of versatile catalysts that can facilitate H_2_ production from these hydrides has become increasingly significant. The generation of efficient catalysts not only enhances the viability of H_2_ as a green energy source but also contributes to the overarching objective of reducing dependence on fossil fuels and mitigating environmental impacts [10,11,12]. The release of H_2_ from hydrogen-rich inorganic hydrides, e.g., sodium borohydride (NaBH_4_) [13,14], ammonium borane (NH_3_BH_3_) [14,15], hydrazine hydrate (N_2_H_4_H_2_O) [16,17], magnesium hydrides (MgH_2_) [18,19], and tetrahydroxy boron (B_2_(OH)_4_) [20,21], has been considered as a feasible, inexpensive, and effective solution to energy and environmental problems. However, fast and controlled H_2_ production from these inorganic hydrides necessitates catalysts that are also non-toxic. Amongst the potential H_2_ carriers, substantial efforts have been made in the design of low-cost and non-noble metal catalysts with a focus on the hydrolysis of NaBH_4_ (Equation (1)) and NH_3_BH_3_ (Equation (2)) [22,23,24,25]. As given in Equations (1) and (2), H_2_ generation from NaBH_4_ and NH_3_BH_3_ only generates non-toxic metaborates.

NaBH_4_ (aq) + 2H_2_O → 4H_2_ (g) + NaBO_2_ (aq) + heat (1)

NH_3_BH_3_ (aq) + 2H_2_O → 3H_2_ (g) + (NH_4_)BO_2_ (aq) + heat (2)

NaBH_4_ has practical advantages such as high gravimetric hydrogen storage capacity (10.8% by weight), chemical stability, room temperature inflammability, and recyclability of hydrolysis by-products [26]. NH_3_BH_3_, on the other hand, is non-toxic, fully soluble, and extremely stable at room temperature with a high hydrogen concentration (19.6% by weight) [27]. Consequently, NaBH_4_ and NH_3_BH_3_ are regarded as the most feasible amongst the chemical hydrogen storage compounds for a range of applications [28,29,30,31]. The non-precious metals such as Co, Ni, and Cu can be readily employed in NaBH_4_ and NH_3_BH_3_ hydrolysis reactions to provide significant cost-saving alternatives in hydrogen generation research for commercial applications in addition to their non-toxic nature [31].

This research complements the findings of Glavee et al., who examined the synthesis of nanoscale particles via the reaction of sodium borohydride (NaBH_4_) with a range of metal salts, including those of cobalt, nickel, iron, and copper [32,33,34,35]. Metal nanoparticles are frequently used as catalysts to enhance/control the reaction rates for many different catalytic reactions. However, the high surface energy of metal nanoparticles tends to cause agglomerates and bigger particles to form, and their ease of oxidation resulting in changes to the surface properties of metal nanoparticles causes an eventual decline in activity. As a result, various materials such as polymeric hydrogels [10], carbon materials [36,37], mesoporous materials [38,39], clay [40,41], and zeolite [42,43] have been employed to stabilize and coat nanoparticles to prevent aggregation and oxidation and/or deactivation.

Therefore, in this investigation, porous carbon particles (PCPs) and their polyethyleneimine (PEI)-modified PCP-PEI forms were used as a template to prepare Co, Ni, and Cu metal nanoparticles in situ as M@PCP and M@PCP-PEI (M:Co, Ni, or Cu), respectively. The prepared M@PCP and M@PCP-PEI composites then were tested as catalysts for the hydrolysis of NaBH_4_ and NH_3_BH_3_ to produce H_2_. The amount of metal nanoparticles was increased via multiple loading/reducing cycles within PCP-based materials. The effects of template, metal species, the amount of metal particle, and temperature on the catalytic activity of the metal catalysts in H_2_ generation reactions from the hydrolysis of NaBH_4_ and NH_3_BH_3_ reaction were studied. The turn-over frequency (TOF, mol H_2_/(mmol cat·min)) and hydrogen generation rate (HGR, mL H_2_/(g cat·min)) values of M@PCP and M@PCP-PEI (M:Co, Ni, or Cu) composite catalysts for the reactions were calculated and compared. Activation energy (Ea), enthalpy (ΔH), and entropy (ΔS) were determined for the Co@PCP-PEI-catalyzed hydrolysis of both NaBH_4_ and NH_3_BH_3_. Moreover, the reuse of Co@PCP-PEI composite catalysts in the hydrolysis of both NaBH_4_ and NH_3_BH_3_ reaction was examined.

## 2. Materials and Methods

### 2.1. Materials

Sucrose (Carlo Erba, Val-de-Reuil, France), tetraethoxysilane (TEOS, 98%, Sigma Aldrich, Milwaukee, WI, USA), ammonium hydroxide (NH_4_OH, 25%, Sigma Aldrich, Milwaukee, WI, USA), and ethanol (ethanol absolute anhydrous, ≥99.9%, Carlo Erba, Cornaredo, Italy) were used for the preparation of porous carbon particles (PCPs). Sulfuric acid (H_2_SO_4_, 95–97%, Merck, Darmstadt, Germany), nitric acid (HNO_3_, ≥65%, Sigma-Aldrich, Milwaukee, WI, USA), dimethylformamide (DMF, 99%, Sigma Aldrich Milwaukee, WI, USA), epichlorohydrin (ECH, 99%, Sigma-Aldrich, Milwaukee, WI, USA), and polyethyleneimine (PEI, 50% in water, M_w_:1800, Sigma-Aldrich, Milwaukee, WI, USA) were used in the modification of PCPs. Cobalt chloride hexahydrate (CoCl_2_.6H_2_O, 98%, Acros, Geel, Belgium), nickel chloride hexahydrate (NiCl_2_.6H_2_O, 98%, Acros, Geel, Belgium), and copper chloride (CuCl_2_ anhydrous, 98%, Acros, Geel, Belgium) were used as corresponding metal ion sources. Sodium borohydride (NaBH_4_, 98%, Merck, Darmstadt, Germany) was used as a reducing agent and for the preparation of metal nanoparticles. Also, both sodium borohydride (NaBH_4_, 98%, Merck) and ammonia–borane (NH_3_BH_3_, 97%, Aldrich, Milwaukee, WI, USA) were used for the production of hydrogen from hydrolysis reactions. Double distilled water was used for washing the prepared particles.

### 2.2. Synthesis and Modification of PCPs

All details about the synthesis of PCPs and modification of PCPs with PEI (PCP-PEI) were reported in the literature in our earlier study [44] and performed accordingly.

### 2.3. In Situ Metal Particle Synthesis Within PCP-PEI

Chloride salts of related metal ions were used in the preparation of Co, Ni, and Cu metal nanoparticles within PCP and PCP-PEI structures. Accordingly, 1.0 g of PCP and PCP-PEI was placed in 250 mL of 1000 ppm aqueous Co(II), Ni(II), and Cu(II) solutions separately, which were mixed at a mixing speed of 500 rpm for 4 h to load the related metal ions into the PCP or PCP-PEI structures. Then, the metal ion-loaded PCP-M(II) and PCP-PEI-M(II) (M:Co, Ni, or Cu) structures were placed separately in a freshly prepared 0.1 M 50 mL aqueous NaBH_4_ solution under a constant mixing speed of 500 rpm. The metal ions were converted to the relevant metal nanoparticles as the reaction was completed, upon which no more gas evolution was observed. Then, these prepared M@PCP and M@PCP-PEI (M:Co, Ni, or Cu) composites were used as catalysts to produce H_2_ from NaBH_4_ and NH_3_BH_3_ hydrolysis reactions.

The amounts of in situ synthesized metal nanoparticles within PCP and PCP-PEI were determined by atomic absorption spectroscopy (Thermo, ICA 3500 AA SPECTRO, Bedford, MA, USA) from the metal ion solution obtained by treating M@PCP and M@PCP-PEI composites with 5 M 20 mL HCl three times for 8 h at a 500 rpm mixing rate to dissolve the metal nanoparticles from the M@PCP and M@PCP-PEI composites.

High-contrast transmission electron microscopy (CTEM, FEI 120 kV, Hillsboro, OR, USA) was utilized to evaluate the morphology and dimensions of in situ synthesized metal nanoparticles within PCP-PEIs. For all transmission electron microscopy (TEM) analyses, M@PCP-PEI particles were initially dispersed in ethanol and subjected to ultrasonic cleaning for a duration of 1.45 min. Subsequently, a drop of the resulting suspension was placed onto a formvar-coated TEM grid and then allowed to dry overnight, and the corresponding images were acquired.

### 2.4. Catalytic Activity of M@PCP and M@PCP-PEI (M:Co, Ni, or Cu) Composites

#### 2.4.1. Hydrolysis of NaBH_4_

After adding certain quantities of M@PCP and M@PCP-PEI (M:Co, Ni, or Cu) composites, which contained the same amount (mmol) of metal particles, 0.0476 mmol M within M@PCP, and 0.0788 mmol M within M@PCP-PEI), were placed in a reaction flask containing 50 mM (0.0965 g) NaBH_4_ in 50 mL distilled water for the hydrolysis of NaBH_4_. The reaction parameters for the hydrolysis reactions were 50 mL 50 mM NaBH_4_ and a mixing rate of 1000 rpm at 30 °C. According to the NaBH_4_ hydrolysis reaction (Equation (1)), the produced H_2_ was recorded as a function of time via a water-filled inverted graded cylinder based on replaced water volume with generated H_2_ gas.

#### 2.4.2. Hydrolysis of NH_3_BH_3_

After adding certain quantities of M@PCP-PEI (M:Co, Ni, or Cu) composites, 0.0788 mmol M) in a reaction flask containing 50 mM (0.0795 g) NH_3_BH_3_ in 50 mL distilled water, the hydrolysis of NH_3_BH_3_ was carried out. The reaction parameters in the hydrolysis reactions were 50 mL 50 mM NH_3_BH_3_ and a mixing rate of 1000 rpm at 30 °C. According to the hydrolysis reaction of NH_3_BH_3_ (Equation (2)), the produced H_2_ was also recorded as a function of time.

### 2.5. Activation Parameters for NaBH_4_ and NH_3_BH_3_ Hydrolysis Catalyzed by Co@PCP-PEI Composites

Activation parameters such as activation energy (Ea), enthalpy (ΔH), and entropy (ΔS) were calculated for the Co@PCP-PEI composite-catalyzed hydrolysis of both NaBH_4_ and NH_3_BH_3_ according to Arrhenius (Equation (3)) and Eyring (Equation (4)) equations.

k = A × e [Ea/RT](3)

ln (k/T) = −(ΔH/R)(1/T) + ln(k_B_/h) + ΔS/R (4)

where k is the reaction rate constant, which was calculated according to a zero-order kinetic expression, E_a_ is the activation energy, T is the absolute temperature (K), k_B_ is the Boltzmann constant (1.381 × 10^−23^ J K^−1^), h is Planck’s constant (6.626 × 10^−34^ J·s), ΔH is the activation enthalpy, ΔS is the entropy, and R is the gas constant (8.314 JK^−1^ mol^−1^).

### 2.6. Reuse of Catalyst in Hydrolysis of NaBH_4_ and NH_3_BH_3_

The reusabilities of Co@PCP-PEI composite catalysts in the hydrolysis of NaBH_4_ and NH_3_BH_3_ were investigated following the literature [12,38]. The reusability parameters, such as conversion% and activity%, of catalysts were compared. The conversion% was defined as the produced amount of hydrogen via the catalyzed reaction according to the stoichiometry of both the hydrolysis of NaBH_4_ and NH_3_BH_3_ as given in Equation (1) and Equation (2), respectively. The activity% was defined as the ratio of the initial H_2_ production rate for each consecutive use based on half the amount of H_2_ that is produced stoichiometrically as the measure of catalyzing efficiency or potency of Co@PCP-PEI composite catalysts for the hydrolysis of NaBH_4_ and NH_3_BH_3_. For the investigation of the reusability of catalysts, after the initial hydrolysis of NaBH_4_ and NH_3_BH_3_, fresh NaBH_4_ and NH_3_BH_3_ at the same quantities as before (0.0965 g for NaBH_4_ and 0.0795 g for NH_3_BH_3_) were added individually nine more times, and the change in the conversion% and activity% of the catalysts were calculated for each use. All the reusability tests of Co@PCP-PEI composite catalysts in the H_2_ production reaction for the hydrolysis of NaBH_4_ and NH_3_BH_3_ were performed in triplicate, and the results of the conversion% and activity% of catalysts were presented as their averages with standard deviations.

## 3. Results and Discussion

### 3.1. Synthesis and Characterization M@PCP and M@PCP-PEI Composite Catalysts

**Figure 1:**
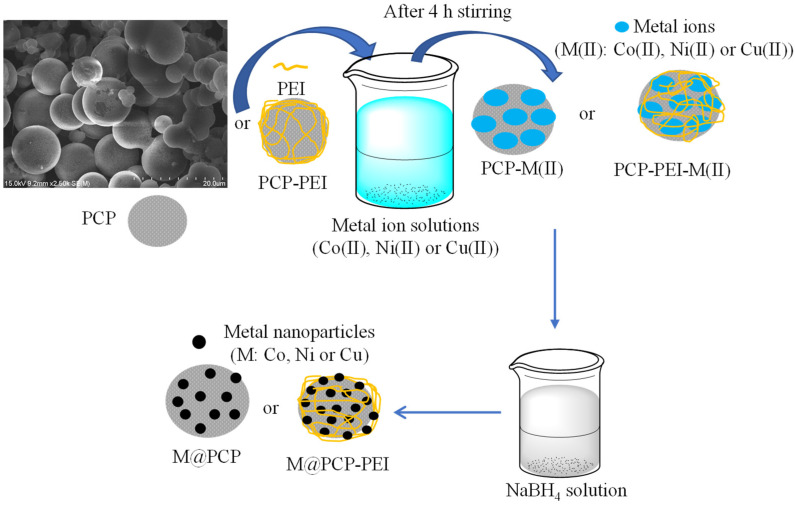
Schematic presentation of Co, Ni, or Cu metal nanoparticle synthesis within PCP and PCP-PEI structures.

The details for the synthesis and characterization of PCP and PCP-PEI structures, which were used here as templates for in situ metal particle preparations, were reported in our previous study [44]. The modification of PCPs with PEI was confirmed with the appearance of −NH_2_ peaks at 1604 cm^−1^ in the FT-IR as well as the change in the surface charge of PCP that was −12.5 ± 2.7 mV and increased to +13.4 ± 3.1 mV after PEI modification [38]. The particle size of PCP particles was reported as 967 ± 61 nm and increased to 1123 ± 92 nm after PEI modification, and the surface area of PCPs decreased from 723 ± 57 m^2^/g to 611 ± 75 m^2^/g upon PEI modification. Moreover, these prepared PCP-PEI structures were used as catalysts in the methanolysis of NaBH_4_ [44]. Here, PCP and PCP-PEI structures were used as a template in the synthesis of metal nanoparticles such as Co, Ni, and Cu nanoparticles in situ as the schematic presentation of the employed process is illustrated in Figure 1.

The PCP and PCP-PEI particles were placed into 1000 ppm 250 mL of Co(II), Ni(II), and Cu(II) metal ion solutions and stirred for 4h at 500 rpm to load the corresponding metal ions into PCP and PCP-PEI structures. Finally, the metal ion-loaded PCP and PCP-PEI structures were placed into 0.1 M 50 mL aqueous NaBH_4_ solutions separately and stirred at 500 rpm until the gas evolution stopped. Then, the obtained M@PCP and M@PCP-PEI (M:Co, Ni, or Cu) composites were used as catalysts for the hydrolysis of both NaBH_4_ and NH_3_BH_3_ to produce H_2_. A comparative analysis of the powder X-ray diffraction (X-RD, Panalytical X’Pert Pro MPD X-Ray Diffractometer, AE Almelo, The Netherlands) patterns for PCP and M@PCP composites is presented in Figure S1. The X-RD data revealed two prominent diffraction peaks at 2θ values of 23.1° (002) and 43.24° (100), which are associated with carbon, as indicated by the PCP X-RD pattern shown in Figure S1 [45,46]. In contrast, the X-RD patterns of the Co@PCP composites exhibited no significant alterations. Conversely, the Ni@PCP composites displayed additional peaks at 2θ = 35.2° (111) and 61.3° (200), which correspond to nickel species within the PCP matrix [47,48]. Furthermore, the X-RD pattern for the Cu@PCP composites indicated several peaks characteristic of copper structures, specifically at 2θ = 36.6°, 42.6°, and 61.8°, corresponding to the (111), (200), and (220) planes of Cu_2_O, respectively [49,50]. Additionally, the peaks at 2θ = 43.7° and 74.1° associated with the (111) and (220) planes were attributed to copper nanoparticles [49,50]. The X-RD patterns for PCP-PEI and the related M@PCP-PEI composites have been documented in previous studies conducted by our research group [51]. To further validate and quantify the in situ synthesis of Co, Ni, and Cu particles within the PCPs, atomic absorption spectroscopy (AAS) analyses were performed, and the quantity of metal nanoparticles were determined. The corresponding results are given in Table 1.

The amount of metal nanoparticles within PCP and PCP-PEI was determined by atomic absorption spectroscopy (AAS) analysis. For this purpose, the metal particle containing the carbon particle composite weighing 100 mg was treated with 5 M 20 mL HCl at room temperature at 500 rpm for 8 h three times. Then, the eluted M(II) ions in the solution were analyzed with AAS. The amounts of M(II) ions were determined with AAS as summarized in Table 1. It was observed that the metal ion contents of M@PCP composites were lower than those of M@PCP-PEI composites, as expected. The presence of amine groups in PCP-PEI led to greater M(II) binding ability due to amine–M(II) complex formation.

The amount of Co metal particles in PCP and PCP-PEI were determined as 19.2 ± 0.9 and 29.8 ± 1.1 mg/g, respectively. On the other hand, Ni contents of Ni@PCP and Ni@PCP-PEI structures were calculated as 13.9 ± 1.0 and 48.2 ± 2.4 mg/g, respectively. Similarly, Cu content in Cu@PCP-PEI was almost 3-fold higher than in Cu@PCP at 31.3 ± 1.9 compared to 90.4 ± 3.2 mg/g.

### 3.2. Catalytic Activity of M@PCP and M@PCP-PEI Composites in Hydrogen Production Reaction from Hydrolysis of NaBH_4_ and NH_3_BH_3_

The catalytic activity of metal-free PCP-PEI structures in the methanolysis of NaBH_4_ was reported earlier in our previous study [44]. Here, the catalytic activity of M@PCP and M@PCP-PEI (M:Co, Ni, or Cu) composite particles for H_2_ production reactions from the hydrolysis of NaBH_4_ and NH_3_BH_3_ were examined. The experimental setup used to determine the catalytic activity of M@PCP and M@PCP-PEI composites was a 50 mL water-filled round bottom flask containing catalysts, and 50 mM NaBH_4_/NH_3_BH_3_. This reaction flask was connected with a trap containing concentrated sulfuric acid that was also connected to the inverted volumetric cylinder filled with water. In this set up, the H_2_ generated in the flask was transferred from the trap to collect any water moisture and then to the water-filled volumetric cylinder. Then, the produced H_2_ was replaced with water in the volumetric cylinder, enabling the easy reading of the volume of produced H_2_.

#### 3.2.1. Hydrogen Production from Hydrolysis of NaBH_4_

**Figure 2:**
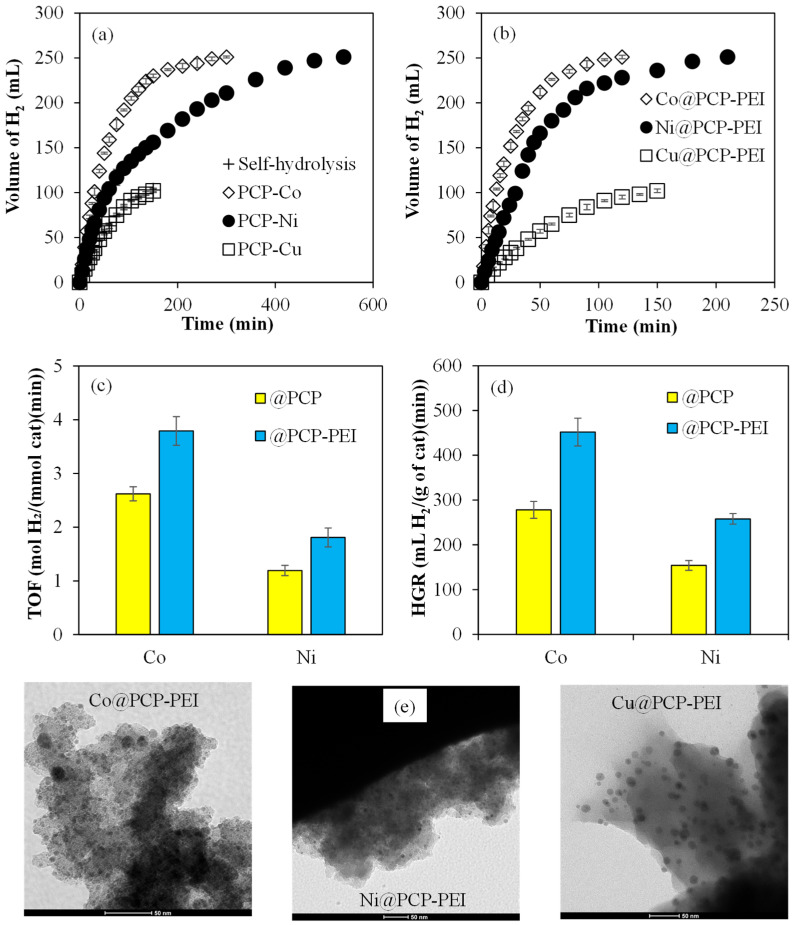
The catalytic activity of (**a**) M@PCP and (**b**) M@PCP-PEI composites on the hydrolysis of NaBH_4_ to produce H_2_; comparison of (**c**) TOF and (**d**) HGR values of M@PCP and M@PCP-PEI composite-catalyzed NaBH_4_ hydrolysis reactions, and (**e**) TEM images of M@PCP-PEI composites [reaction conditions: M:Co, Ni, or Cu, 0.0476 mmol M for M@PCP, 0.0788 mmol M for M@PCP-PEI, 50 mL water, 0.0965 g NaBH4, 30 °C, 1000 rpm].

The catalytic activities of M@PCP and M@PCP-PEI (M:Co, Ni, or Cu) composites in the hydrolysis of NaBH_4_ were compared and the related graphs are given in Figure 2. To compare the catalytic activities of M@PCP composites in the NaBH_4_ hydrolysis reaction, 156 mg of Co@PCP, 200 mg of Ni@PCP, and 96 mg of Cu@PCP composites, with all having around 0.048 mmol metal nanoparticles, were used. It can be clearly seen in Figure 2a that the Co@PCP composites catalyzed the hydrolysis of NaBH_4_ completely in 300 min with 251 ± 1 mL of H_2_ production. On the other hand, Ni@PCP composites catalyzed the same reaction completely in 540 min with 251 ± 1 mL of H_2_ produced. However, Cu@PCP composites did not exhibit any catalytic activity for NaBH_4_ hydrolysis as only 100 mL of H_2_ was produced in 150 min, which is equal to the amount of produced H_2_ from the self-hydrolysis reaction of NaBH_4_ (without catalyst) in 150 min. Moreover, the catalytic activities of M@PCP-PEI (M:Co, Ni, or Cu) for NaBH_4_ hydrolysis were also compared and the results are presented in Figure 2b. For this objective, 156 mg of Co@PCP-PEI, which equates to 0.0788 mmol metal particles, and equal mmol metal particles containing 96 mg Ni@PCP-PEI and 55 mg Cu@PCP-PEI composites were used for the catalytic hydrolysis of NaBH_4_. As clearly seen, both Co@PCP-PEI and Ni@PCP-PEI composites catalyzed the reactions much faster than Co@PCP and Ni@PCP composites. The hydrolysis of NaBH_4_ catalyzed by Co@PCP-PEI and Ni@PCP-PEI composites were completed in 120 and 210 min, respectively, with both producing 251 ± 1 mL of H_2_. As the amounts of metal nanoparticles are higher within Co@PCP-PEI and Ni@PCP-PEI composites compared to Co@PCP and Ni@PCP composites, these results are reasonable.

For the comparison of the catalytic activity of M@PCP and M@PCP-PEI composite catalysts, important parameters such as turn-over frequency (TOF, mol H_2_/(mmol cat·min)) and hydrogen generation rate (HGR, mL H_2_/(g cat·min)) for M@PCP and M@PCP-PEI composites were calculated and are illustrated in Figure 2c,d, respectively. For both TOF and HGR calculations, the number of catalysts (moles) was taken into consideration as the particles have an approximate 10 nm size range, assuming most metal nanoparticles possess many active sites in the composite systems and 100% are active. In Figure 2c, the calculated TOF values for both Co@PCP-PEI and Ni@PCP-PEI are 3.8 ± 0.3 and 1.8 ± 0.2 mol H_2_/(mmol cat·min), respectively. These are almost 1.5-fold higher than the values for Co@PCP and Ni@PCP, which are 1.6 ± 0.1 and 1.2 ± 0.1 (mol H_2_/(mmol cat·min)), respectively. The calculated HGR values for the M@PCP and M@PCP-PEI composite-catalyzed hydrolysis of NaBH_4_ are shown in Figure 2d. The HGR values of 452 ± 31 for Co@PCP-PEI and 258 ± 12 mL H_2_/(g cat·min) for Ni@PCP-PEI are higher than the values for Co@PCP and Ni@PCP which are 278 ± 19 and 154 ± 11 mL H_2_/(g cat·min), respectively. The effects of the amount of metal particle and temperature on the catalytic activity of the Co@PCP-PEI catalyst in the hydrolysis of NaBH_4_ were also investigated because of the higher TOF and HGR values among the prepared catalysts used in the hydrolysis of NaBH_4_. Moreover, to confirm the presence of in situ synthesized metal nanoparticles within PCP-based materials and their sizes, TEM images of M@PCP-PEI composites with higher metal nanoparticle content and catalytic activity were taken and are given in Figure 2e. The dimensions of in situ synthesized Co and Ni nanoparticles within PCP-PEI are about 5 and 10 nm, whereas Cu nanoparticles exhibited slightly bigger particles sizes varying from 10 to 20 nm.

**Figure 3:**
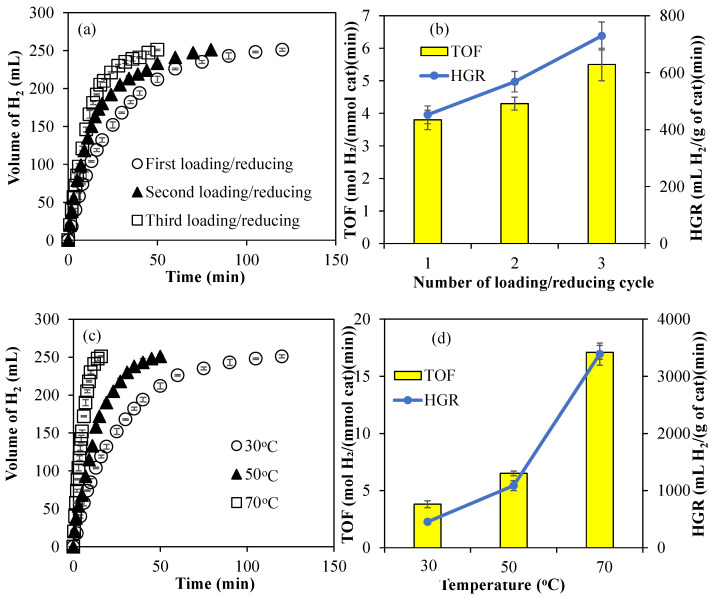
(**a**) The effect of the amount of Co metal nanoparticles on the catalytic activity of Co@PCP-PEI composites in the hydrolysis of NaBH_4_; (**b**) comparison of TOF and HGR values of multiple-Co(II)-loaded/reduced Co@PCP-PEI composite catalyst; (**c**) effect of temperature on the hydrolysis of NaBH_4_ catalyzed by Co@PCP-PEI composite catalysts (Co: 29.8 ± 1.1 mg/g); (**d**) comparison of TOF and HGR values of Co@PCP-PEI composite catalysts for the hydrolysis of NaBH_4_ carried out at different temperatures (Co: 29.8 ± 1.1 mg/g) [reaction conditions: 50 mL water, 0.0965 g NaBH_4_, mixing rate: 1000 rpm].

The amounts of Co metal particles within Co@PCP-PEI composites were increased by multiple loading and reducing cycles. For example, after Co metal nanoparticles were synthesized within PCP-PEI as Co@PCP-PEI, these composites were then placed in 250 mL 1000 ppm aqueous Co(II) ion solutions and stirred for 4 h for the second loading of Co(II) ions into Co@PCP-PEI composites. Then, these two-time Co(II) ion-loaded Co@PCP-PEI composites were washed with water to remove unbound Co(II) ions on the surfaces, then placed into a freshly prepared 50 mL 0.1 M NaBH_4_ solution to reduce for a second time the loaded Co(II) ions to Co metal nanoparticles, and stirred at 500 rpm until the evolution of the gas stopped as an indication of the reduction of Co(II) to the corresponding Co metal nanoparticles. This reloading/reducing cycle was repeated one more time for the preparation Co@PCP-PEI composites. The amounts of Co metal particles in Co@PCP-PEI composites after the first, second, and third loading/reducing process were determined as 29.8 ± 1.1, 35.6 ± 2.2, and 44.3 ± 4.9 mg/g, respectively, and are given in Table 1. It is obvious that the amount of metal nanoparticles within PCP-PEI can easily increase via multiple loading/reducing cycles. The effects of the amount of Co metal particles within the Co@PCP-PEI composite on its catalytic activity were also investigated and the results are given in Figure 3a. The catalytic activity of one-time loaded/reduced Co@PCP-PEI composite-catalyzed hydrolysis of NaBH_4_ was completed in 120 min with 251 ± 1 mL of H_2_ production, whereas the same amount of H_2_ was produced in 80 and 50 min after the second and third Co(II) ion-loaded/reduced cycles. The comparison of TOF and HGR values for multiple loaded/reduced Co@PCP-PEI composite-catalyzed reactions is shown in Figure 3b. The calculated TOF values for Co@PCP-PEI composite-catalyzed reactions increased from 3.8 ± 0.3 mol H_2_/(mmol cat·min) to 5.5 ± 0.5 mol H_2_/(mmol cat·min) with increasing loading/reducing cycles of Co@PCP-PEI catalysts. In addition, the calculated HGR values also increased from 452 ± 31 mL H_2_/(g cat·min) to 729 ± 49 mL H_2_/(g cat·min) for the Co@PCP-PEI-catalyzed reaction from one cycle to three. The increase in the amount of Co metal nanoparticles within the Co@PCP-PEI composite catalysts also led to an increase in catalytic activity for the hydrolysis of NaBH_4_, which exhibited higher TOF and HGR values.

The effect of temperature on the catalytic activity of the Co@PCP-PEI-catalyzed hydrolysis of NaBH_4_ was also investigated by carrying the catalyzed reactions at 30, 50, and 70 °C. In Figure 3c, the Co@PCP-PEI-catalyzed hydrolysis of NaBH_4_ at 30, 50, and 70 °C was completed in 120, 50, and 16 min, respectively each with the same amount of H_2_ produced, 251 ± 1 mL. The reaction rates for the Co@PCP-PEI-catalyzed hydrolysis of NaBH_4_ was increased with the increase in the temperature, as expected. Additionally, as demonstrated in Figure 3d, the TOF and HGR values of the Co@PCP-PEI-catalyzed hydrolysis of NaBH_4_ also increased with the increase in reaction temperature from 30 to 70 °C. The TOF value for the Co@PCP-PEI-catalyzed reaction, 3.8 ± 0.3 mol H_2_/(mmol cat·min) at 30 °C, was increased almost 5-fold by increasing the temperature to 70 °C with a 17.1 ± 0.6 mol H_2_/(mmol cat·min) TOF value. Similarly, the HGR values of the Co@PCP-PEI-catalyzed hydrolysis of NaBH_4_ at 30 °C was increased by almost 8-fold at 70 °C, i.e., from 452 ± 31 to 3390 ± 193 mL H_2_/(g cat·min). The HGR values obtained at 30 °C for NaBH_4_ hydrolysis reactions utilizing Co@PCP-PEI composites are comparatively lower than those reported for other catalysts containing Co metal nanoparticles reported in the literature, e.g., the NaBH_4_ hydrolysis reaction catalyzed by B-doped Co_3_O_4_ nanowires with 7055 mL H_2_/(g cat·min) [52], nitrogen-doped mesoporous graphitic carbon-encapsulated cobalt nanoparticles (Co@NMGC) with 3575 mL H_2_/(g cat·min) [53], bacterial cellulose/Co-B (BC/Co-B) nanocomposites with 3887 mL H_2_/(g cat·min) [54], Co nanoparticles supported on carbon nanospheres (CNSs) (CNSs@Co) with 7447 mL H_2_/(g cat·min) [55], Co-CeOx/nitrogen-doped carbon nanosheet (NCNS) with 28,410 mL H_2_/(g cat·min) [56], CoB/TiO_2−*x*_ catalyst with 3070 mL H_2_/(g cat·min) [57], Co_6_FeAl-LDH catalyst with 4955 mL H_2_/(g cat·min) [58], Co(30%)/Fe_3_O_4_@GO with 6005 mL H_2_/(g cat·min) [59], and g-C_3_N_4_/Co–Mo–B/Ni foam with 9958 mL H_2_/(g cat·min) values [60]. Nevertheless, these composites revealed promising potential to perform competitively at elevated temperatures. It is important to acknowledge that the necessity for high operational temperatures may pose economic and energy-related challenges for the synthesized catalyst. Furthermore, an increase in the concentration of Co nanoparticles incorporated into the PCP-PEI matrix is associated with an improvement in the HGR value. Therefore, this limitation can be mitigated by increasing the concentration of Co nanoparticles within the PCP-PEI composites.

#### 3.2.2. H_2_ Production for Hydrolysis of NH_3_BH_3_

**Figure 4:**
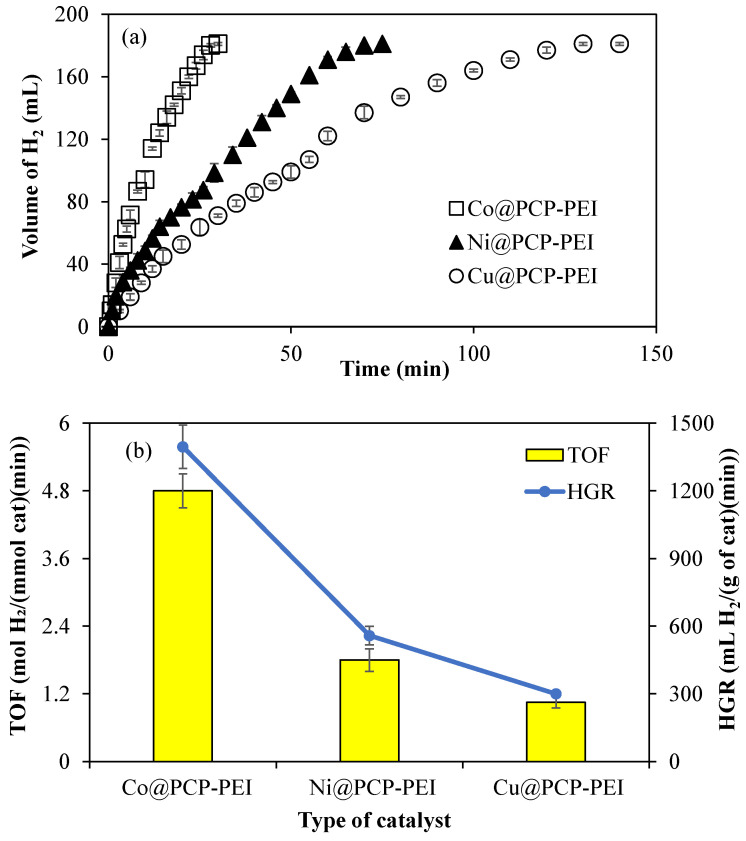
(**a**) The catalytic activity of M@PCP-PEI composite catalysts in the hydrolysis of NH_3_BH_3_ to produce H_2_, and (**b**) comparison of TOF and HGR values of the M@PCP-PEI composite catalyst [reaction condition: M:Co, Ni, or Cu, 0.0788 mmol M, 50 mL water, 0.0795 g NH_3_BH_3_, 30 °C, 1000 rpm].

Another H_2_ carrier, NH_3_BH_3_, can also be catalyzed by M@PCP-PEI (M:Co, Ni, or Cu) composites to produce H_2_. Therefore, M@PCP-PEI (M:Co, Ni, or Cu) composites were used as a catalyst in the hydrolysis of NH_3_BH_3_. As illustrated in Figure 4a, the catalytic performance of the M@PCP-PEI (M:Co, Ni, or Cu) composite catalyst was compared using 156 mg of Co@PCP-PEI, 96 mg of Ni@PCP-PEI, and 55 mg of Cu@PCP-PEI composites, which refers to 0.0788 mmol metal nanoparticles in the hydrolysis of NH_3_BH_3_. Co@PCP-PEI composites catalyzed the complete hydrolysis of NH_3_BH_3_ in 28 min with 181 ± 1 mL H_2_ production, which was faster than Ni@PCP-PEI and Cu@PCP-PEI composite-catalyzed reactions, which were completed in 70 and 130 min, respectively, with 181 ± 1 mL H_2_ production.

The comparison of TOF and HGR values for the M@PCP-PEI (M:Co, Ni, or Cu)-catalyzed hydrolysis of NH_3_BH_3_ is given in Figure 4b. It is obvious that Co@PCP-PEI composites exhibited higher TOF and HGR values, at 4.8 ± 0.3 mol H_2_/(mmol cat·min) and 1395 ± 96 mL H_2_/(g cat·min), respectively, than the other two composite catalysts. These TOF and HGR values calculated for Co@PCP-PEI catalyzed reactions are almost 2- and 4-fold higher than the calculated TOF and HGR values for Ni@PCP-PEI and Cu@PCP-PEI composite-catalyzed reactions, respectively.

**Figure 5:**
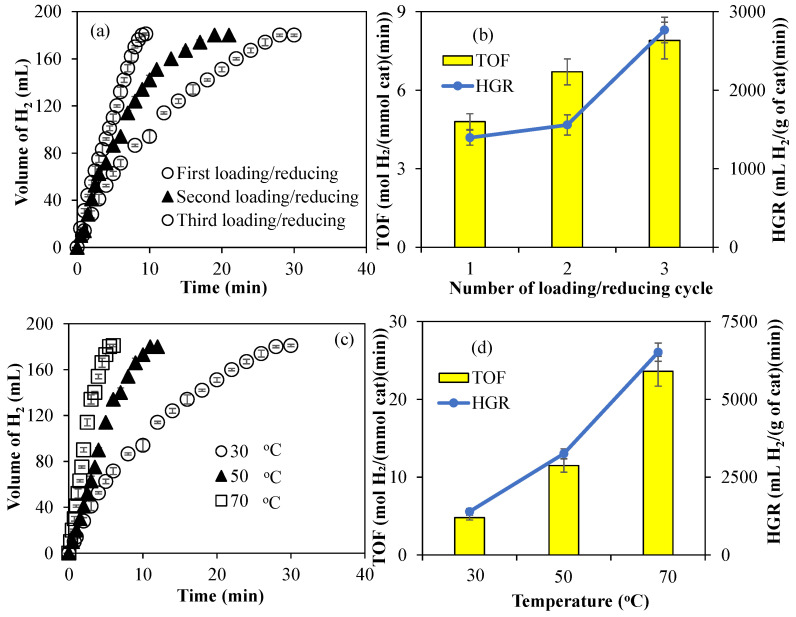
(**a**) The effect of the amounts of Co metal nanoparticles on the catalytic activity of Co@PCP-PEI composites in the hydrolysis of NH_3_BH_3_; (**b**) comparison of TOF and HGR values; (**c**) the effect of temperature on the Co@PCP-PEI composite-catalyzed hydrolysis of NH_3_BH_3_; (**d**) comparison of TOF and HGR values at different temperatures [reaction conditions: 50 mL water, 0.07955 g NH_3_BH_3_, 1000 rpm].

The effects of the amount of metal nanoparticles and the reaction temperature on the hydrolysis of NH_3_BH_3_ were investigated for Co@PCP-PEI composite catalysts due to their higher TOF and HGR values. As presented in Figure 5a, the effect of the amounts of Co nanoparticles within PCP-PEI is increased with multiple loading/reducing cycles, as mentioned previously. The catalytic activity of Co@PCP-PEI composites in the hydrolysis of NH_3_BH_3_ was increased with the increase in the amount of Co metal nanoparticles. The Co@PCP-PEI composite-catalyzed hydrolysis of NH_3_BH_3_ was completed in 28 min with 181 ± 1 mL H_2_ production, whereas the same reaction was completed in 21 and 9.5 min, respectively, for two- and three-time Co(II)-loaded/reduced Co@PCP-PEI composites as the catalyst, with each producing 181 ± 1 mL H_2_. The comparison of TOF and HGR values of the one-, two-, and three-time Co(II) ion-loaded/reduced composite-catalyzed hydrolysis of NH_3_BH_3_ also revealed that these values increased with the increase in amount of Co metal nanoparticles (or increased number of loaded/reduced cycles), as shown in Figure 5b. The TOF value of the one-time Co(II) ion-loaded/reduced CP-PEI, Co@PCP-PEI composite-catalyzed hydrolysis of NH_3_BH_3_ is 4.8 ± 0.3 mol H_2_/(mmol cat·min) and increased to 7.9 ± 0.3 mol H_2_/(mmol cat·min) upon using three-time Co(II) ion-loaded/reduced Co@PCP-PEI composite catalysts. Similarly, the HGR values were calculated for first- and third-time Co(II) ion-loaded Co@PCP-PEI composites that were used in the hydrolysis of NH_3_BH_3_ and were calculated as 1395 ± 96 and 2766 ± 162 mL H_2_/(g cat·min), respectively. The calculated TOF and HGR values for the first-time Co(II) ion-loaded/reduced composite catalyst were increased almost 2-fold upon three-time Co(II) ion-loaded/reduced cycles for Co@PCP-PEI composite-catalyzed reactions. This is reasonable as the increased amount of Co metal particles affords higher catalytic performance than lesser amounts of Co metal particle-containing Co@PCP-PEI composite catalysts. On the other hand, it can be clearly seen from Figure 5c that the increase in the reaction temperature of the Co@PCP-PEI-catalyzed hydrolysis of NH_3_BH_3_ increased the reaction rates as anticipated. The hydrolysis of NH_3_BH_3_ was completed in 28, 12, and 6 min in the presence of the Co@PCP-PEI catalyst at 30, 50, and 70 °C, respectively, with all producing 181 ± 1 mL H_2_.

It is also evident from the comparison of TOF and HGR values as illustrated in Figure 5d for the Co@PCP-PEI-catalyzed hydrolysis of NH_3_BH_3_ at 30, 50, and 70 °C that the increase in the reaction temperature increases the values of TOF and HGR. The TOF value of 4.8 ± 0.3 mol H_2_/(mmol cat·min) and the HGR value of 1395 ± 96 mL H_2_/(g cat·min) for the Co@PCP-PEI-catalyzed hydrolysis of NH_3_BH_3_ at 30 °C were increased almost 5-fold and calculated as 23.6 ± 0.3 mol H_2_/(mmol cat·min) (TOF value) and 6514 ± 293 mL H_2_/(g cat·min) (HGR value), respectively at 70 °C. The calculated HGR values for the Co@PCP-PEI-catalyzed NH_3_BH_3_ hydrolysis reaction are already competitive with those of similar studies reported in the literature such as those of the Co–P/Ni foam-catalyzed hydrolysis of NH_3_BH_3_ with 1248 mL H_2_/(g cat·min) [61] and CoB nanowire-catalyzed hydrolysis of NH_3_BH_3_ with 2667 mL H_2_/(g cat·min) [62].

### 3.3. Activation Parameters for Co@PCP-PEI-Catalyzed Hydrolysis of Both NaBH_4_ and NH_3_BH_3_

The activation energy (Ea), enthalpy (ΔH) and entropy (ΔS) for the Co@PCP-PEI-catalyzed hydrolysis of NaBH_4_ and NH_3_BH_3_ were calculated using Arrhenius and Eyring equations from the half H_2_ production curves with time at 30, 50, and 70 °C. The corresponding Arrhenius and Eyring plots of the Co@PCP-PEI-catalyzed hydrolysis of both NaBH_4_ and NH_3_BH_3_ are given in Figure S2. From this figure, the calculated Ea, ΔH, and ΔS are summarized in Table 2.

The Ea values for the Co@PCP-PEI=catalyzed hydrolysis of both NaBH_4_ and NH_3_BH_3_ were calculated as 29.5 and 32.3 kJ/mol, respectively. The Ea value of the Co@PCP-PEI-catalyzed hydrolysis of NaBH_4_ was compared with those of similar studies reported in the literature; these varied, with most being higher. For example, NaBH_4_ hydrolysis reaction were catalyzed by B-doped Co_3_O_4_ nanowires with Ea = 29.7 kJ/mol [52], nitrogen-doped mesoporous graphitic carbon-encapsulated cobalt nanoparticles (Co@NMGC) with Ea = 35.2 kJ/mol [53], bacterial cellulose/Co-B (BC/Co-B) nanocomposites with Ea = 56.4 kJ/mol [54], Co nanoparticles supported on carbon nanospheres (CNSs) (CNSs@Co) with Ea = 40.8 kJ/mol [55], Co-CeOx/nitrogen-doped carbon nanosheet (NCNS) with Ea = 44.2 kJ/mol [56], CoB/TiO_2-*x*_ catalyst with Ea = 57.0 kJ/mol [57], Co_6_FeAl-LDH catalyst with Ea = 35.5 kJ/mol [58], Co(30%)/Fe_3_O_4_@GO with Ea = 44.4 kJ/mol [59], and g-C_3_N_4_/Co–Mo–B/Ni foam with Ea = 52.6 kJ/mol [60]. In this study, the Ea value (29.5 kJ/mol) is lower than the reported energy activation values of the last two years of the Co-based catalysts, as presented in Table 2. On the other hand, the determined Ea value for the Co@PCP-PEI-catalyzed hydrolysis of NH_3_BH_3_, 32.3 kJ/mol, is also competitive with reported activation energy values for the same reaction reported in the literature, such as the Co–Mo–B/Ni foam-catalyzed hydrolysis of NH_3_BH_3_ with Ea = 44.3 kJ/mol [61], Co–P/Ni foam-catalyzed hydrolysis of NH_3_BH_3_ with Ea = 48.0 kJ/mol [62], CoB nanowire-catalyzed hydrolysis of NH_3_BH_3_ with Ea = 16.2 kJ/mol [63], Ag@Pd composite-catalyzed hydrolysis of NH_3_BH_3_ with Ea = 50.1 kJ/mol [64], and Ru_1_Ni_1.90_/nitrogen-doped carbon skeleton (NCS)-catalyzed hydrolysis of NH_3_BH_3_ with Ea = 26.5 kJ/mol [65]. Therefore, it is apparent that Co@PCP-PEI composite catalysts are more favorable materials in terms of H_2_ generation using either of the H_2_ sources, NaBH_4_ and NH_3_BH_3_.

### 3.4. Reusability of Co@PCP-PEI Composite Catalyst

**Figure 6:**
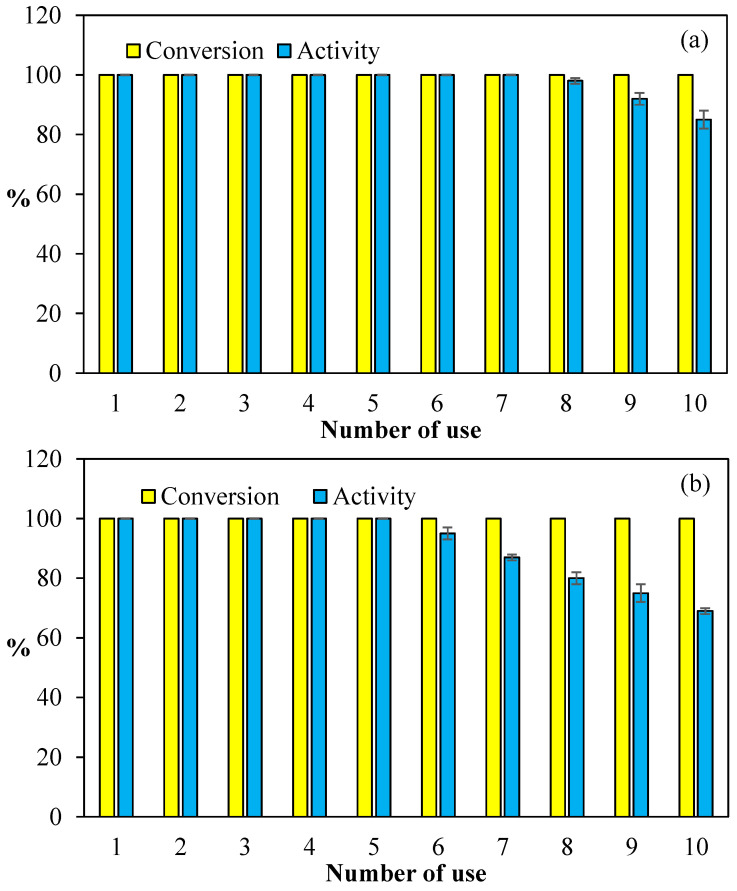
The reusability of Co@PCP-PEI composite catalysts in the hydrolysis of (**a**) NaBH_4_ and (**b**) NH_3_BH_3_ [reaction conditions: 0.0788 mmol Co, 50 mL water, 0.0965 g NaBH_4_, 0.0795 g NH_3_BH_3_, 30 °C, 1000 rpm].

The cost consideration of catalysts in industrial applications is one of the most important constraints. The reusability of catalysts to reduce costs in industrial applications is of paramount significance. Therefore, the reusability of Co@PCP-PEI composites in both hydrolysis reactions of NaBH_4_ and NH_3_BH_3_ were tested, and the corresponding graphs are given in Figure 6. In Figure 6a, the reuse of Co@PCP-PEI composite catalysts in the hydrolysis of NaBH_4_ is given and 100% conversions were attained for all hydrolyses of NaBH_4_ up to 10 consecutive uses. On the other hand, the activity% of the Co@PCP-PEI composite catalyst for the hydrolysis of NaBH_4_ in 10 consecutive usages revealed that the activity remains at 100% for up to 7 consecutive uses, and after the 10th use, approximately 85% of its activity is preserved.

Additionally, the reusability of Co@PCP-PEI composite catalysts in the hydrolysis of NH_3_BH_3_ was also compared and the results are shown in Figure 6b. As presented, the Co@PCP-PEI composite-catalyzed hydrolysis of NH_3_BH_3_ afford 100% conversion even at the 10th consecutive use. On the other hand, the activity of this catalyst maintained its activity% up to the 5th use at 100%, and slowly decreased, e.g., between the 6th-10th use, the activity% was reduced from 95 ± 2 to 69 ± 1%. Nevertheless, the Co@PCP-PEI composite catalysts exhibited almost 70% activity at the 10th repetitive use.

Overall, the seven-time and 5-time successive uses of Co@PCP-PEI composite catalysts afford 100% activity in the hydrolysis of NaBH_4_ and NH_3_BH_3_, standing out as the most important feature of these catalysts along with their 100% conversion capability with up to 10 repeated uses, making this catalyst system a promising material for industrial applications. The observed reduction in the catalytic activity% of Co@PCP-PEI during the hydrolysis reactions of NaBH_4_ and NH_3_BH_3_ is due to the accumulation of reaction by-products on the catalyst surface. This phenomenon has been reported in the existing literature and was confirmed with XRD and FT-IR analyses [66,67].

## 4. Conclusions

PCP and PCP-PEI structures were successfully used as templates to prepare metal nanoparticles such as Co, Ni, and Cu, in situ. The prepared M@PCP and M@PCP-PEI (M:Co, Ni, or Cu) composites were used as catalysts for the hydrolysis of both NaBH_4_ and NH_3_BH_3_ to produce H_2_. The hydrolysis of NaBH_4_ and NH_3_BH_3_ catalyzed by Co@PCP-PEI resulted in higher TOF and HGR values than the M@PCP and M@PCP-PEI (M:Ni or Cu) composite catalysts. The TOF values for the Co@PCP-PEI composite-catalyzed hydrolysis of NaBH_4_ and hydrolysis of NH_3_BH_3_ were calculated as 3.8 ± 0.3 and 4.8 ± 0.3 mol H_2_/(mmol cat·min), respectively; the HGR values were calculated as 452 ± 31 and 1395 ± 96 mL H_2_/(g cat·min), in the same order. Moreover, the determined Ea value for the Co@PCP-PEI composite-catalyzed hydrolysis of NaBH_4_ was 29.3 kJ/mol, which is lower than the Ea value of the Co@PCP-PEI composite-catalyzed hydrolysis of NH_3_BH_3_, which was 32.5 kJ/mol. However, these Ea values are competitive with those of similar reported studies in literature. It was further demonstrated that the Co@PCP-PEI composite possesses high reuse capability, with 100% conversions up to 10 successive uses in the hydrolysis of NaBH_4_ and NH_3_BH_3_. After seven and five repetitive deployments, 100% of the activities were obtained and there was a slight reduction afterwards. As a result, M@PCP-PEI (M:Co, Ni, and Cu) catalyst systems with transition metal nanoparticles can be presumed economically viable and may be employed in sophisticated H_2_-driven devices for clean and environmentally benign applications. A significant finding of the relevant research is its contribution for the development of multifunctional materials that can also be used in other applications including the catalytic reduction of even CO_2_ while simultaneously adsorbing it, e.g., M@PCP or M@PCP-PEI were reported for these purposes [51,68]. These materials provide multiple advantages to address many issues beyond renewable energy sources contributing to mitigating global warming.

## Figures and Tables

**Table 1:** The amounts of Co, Ni, and Cu metal nanoparticles within PCP-based structures.

Composite	Amount of Metal Nanoparticles (mg/g)		
	Co	Ni	Cu
@PCP	19.2 ± 0.9	13.9 ± 1.0	31.3 ± 1.9
@PCP-PEI	29.8 ± 1.1	48.2 ± 2.4	90.4 ± 3.2
Number of loading/reducing cycle			
	1st	2nd	3rd
Co@PCP-PEI	29.8 ± 1.1	35.6 ± 2.2	44.3 ± 4.9

**Table 2:** The Ea, ΔH, and ΔS values for the Co@PCP-PEI composite-catalyzed hydrolysis of NaBH_4_ and NH_3_BH_3_ and their comparison with some similar studies reported in the literature.

Catalyst	Hydrolysis Reaction of	Activation Parameters			[REF]
		Ea (kJ/mol)	ΔH (kJ/mol)	ΔS (J/mol.K)	
Co@PCP	NaBH_4_	29.3	26.1	−182.9	This study
B-doped Co_3_O_4_	NaBH_4_	29.7	-	-	[52]
Co@NMGC	NaBH_4_	35.2	-	-	[53]
BC/Co-B	NaBH_4_	56.4	-	-	[54]
CNSs@Co	NaBH_4_	40.8	-	-	[55]
Co-CeOx/NCNS	NaBH_4_	44.2	-	-	[56]
CoB/TiO_2_-x	NaBH_4_	57.0	-	-	[57]
Co_6_FeAl-LDH	NaBH_4_	35.5	-	-	[58]
Co(30%)/Fe_3_O_4_@GO	NaBH_4_	44.4	-	-	[59]
g-C_3_N_4_/Co–Mo–B/Ni	NaBH_4_	52.6	-	-	[60]
Co@PCP	NH_3_BH_3_	32.5	29.2	−196.3	This study
Co–P/Ni	NH_3_BH_3_	48.0	-	-	[61]
CoB	NH_3_BH_3_	16.2	-	-	[62]
Co–Mo–B/Ni	NH_3_BH_3_	44.3	-	-	[63]
Ag@Pd	NH_3_BH_3_	50.1	-	-	[64]
Ru1Ni1.90/NCS	NH_3_BH_3_	26.5	-	-	[65]

## Data Availability

All the data generated in this research is retained within the manuscript and in Supplementary Materials.

## Supplementary Materials

The following supporting information can be downloaded at: https://www.mdpi.com/article/10.3390/mi16020172/s1, Figure S1: The XRD patterns of M@PCP (M:Co, Ni, or Cu) composites.; Figure S2: The Arrhenius graphs of the hydrolysis of (a) NaBH_4_ and (b) NH_3_BH_3_, and Eyring graphs of the hydrolysis of (c) NaBH_4_ and (d) NH_3_BH_3_ catalyzed by Co@PCP-PEI composites.
