# Supplementary material for: Hydrogen Production from Chemical Hydrides via Porous Carbon Particle Composite Catalyst Embedding of Metal Nanoparticles
Source: Micromachines (Basel). 2025 Jan 31;16(2):172. doi: 10.3390/mi16020172 (PMC11857494; doi:10.3390/mi16020172)
Supplement: Supplementary file 1 [file micromachines-16-00172-s001.zip › micromachines-3425605-supplementary.pdf]

Hydrogen production from chemical hydrides via porous carbon particles composite catalysts embedding metal nanoparticles

Sahin Demirci[1], Osman Polat[2], Nurettin Sahiner[2,3,4,5*]

[1] Department of Food Engineering, Faculty of Engineering, Istanbul Aydin University, Florya Halit Aydin Campus, Istanbul 34295, Turkey
[2] Department of Chemical and Biomolecular Engineering, University of South Florida, Tampa, FL, 33620, USA.
[3] Department of Chemistry, Faculty of Sciences, Canakkale Onsekiz Mart University, Terzioglu Campus, Canakkale, 17100, Turkey.
[4] Department of Ophthalmology, Morsani College of Medicine, University of South Florida, 12901 Bruce B. Downs Blvd, MDC21, Tampa, FL 33612, USA.
[5] Department of Bioengineering, U.A. Whittaker College of Engineering, Florida Gulf Coast University, Fort Myers, FL 33965, USA. (sahiner71@gmail.com ; nashiner@fgcu.edu)

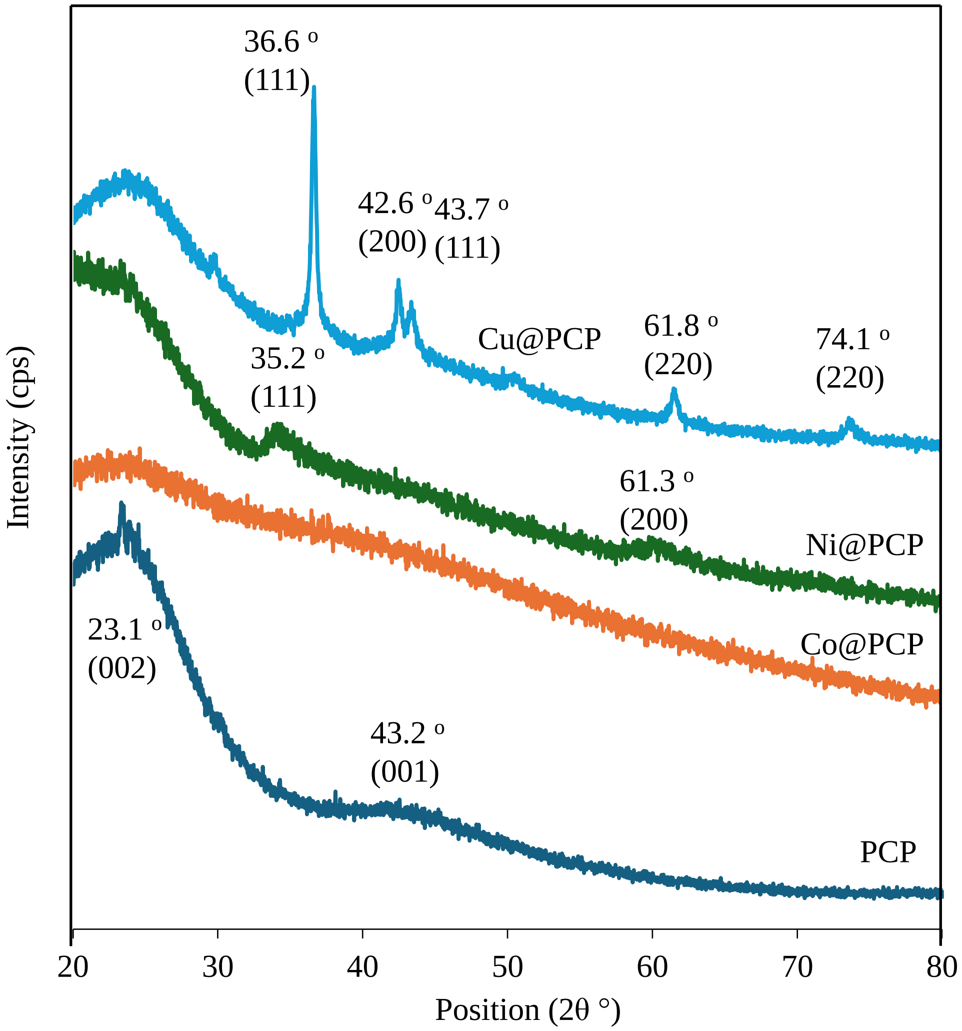

Figure S1. The X-RD patterns of M@PCP (M: Co, Ni, or Cu) composites.

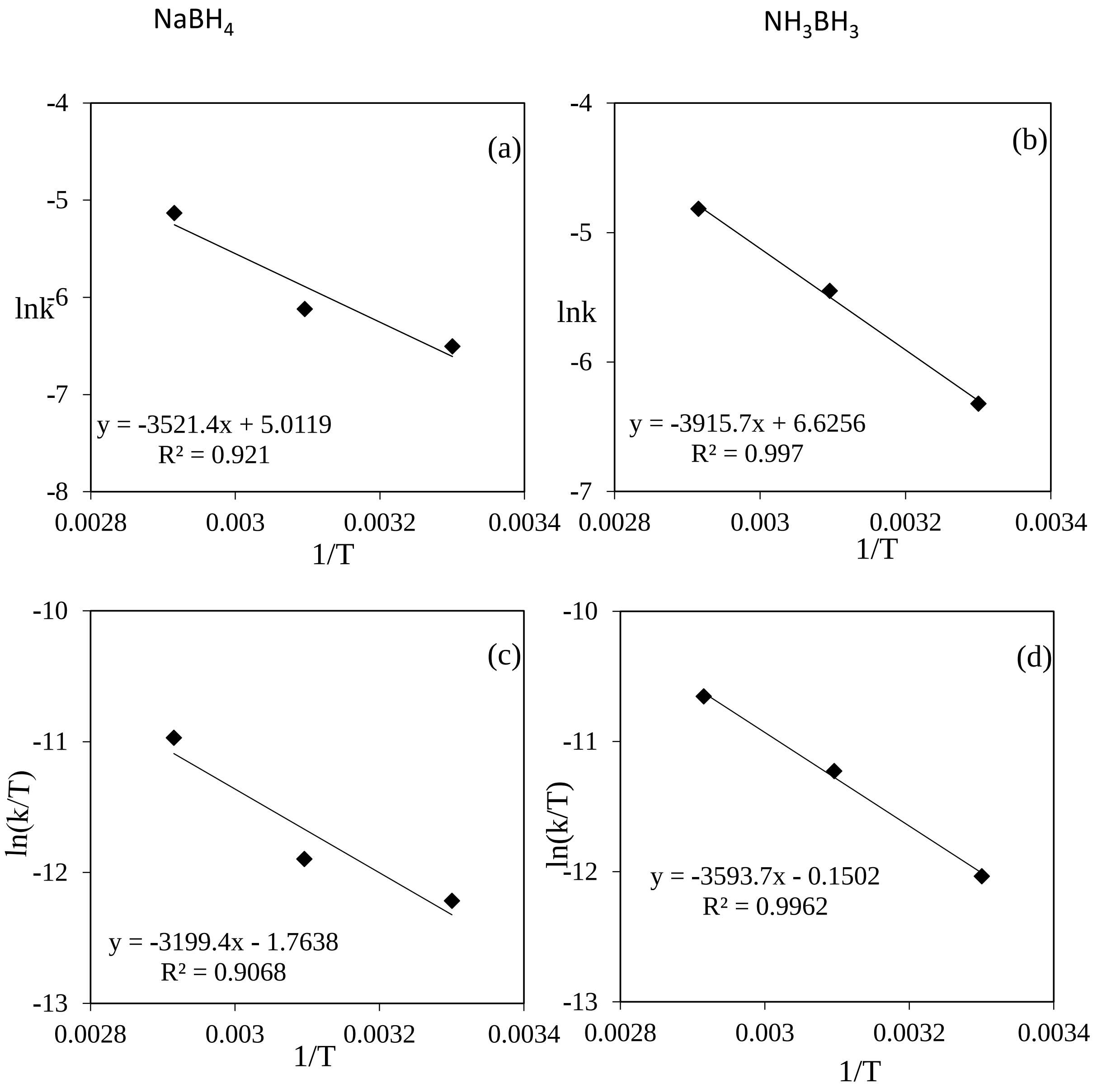

Figure S2. The Arrhenius graphs of hydrolysis of (a) $NaBH_4$, (b) NH_3BH_3, and Eyring graphs of hydrolysis of (c) $NaBH_4$, (d) NH_3BH_3 reaction catalyzed by Co@PCP-PEI composites.
